# May

**Published:** 1890-05

**Authors:** 


FROM THE GERMAN OF HEINRICH HEINE.

*Translated for Hall's Journal of Health.*

In the charming month of May,
When all the buds are swelling,
To my heart there comes alway,
Love all else dispelling.

In the charming month of May,
When all the birds are singing,
Doth my heart confess to thee,
Every wish revealing.

